# Evaluation of antibiotic resistance, toxin-antitoxin systems, virulence factors, biofilm-forming strength and genetic linkage of *Escherichia coli* strains isolated from bloodstream infections of leukemia patients

**DOI:** 10.1186/s12866-023-03081-8

**Published:** 2023-11-04

**Authors:** Mahdaneh Roshani, Mohammad Taheri, Alireza Goodarzi, Rassoul Yosefimashouf, Leili Shokoohizadeh

**Affiliations:** 1 Department of Microbiology, Faculty of Medicine, Hamadan University of Medical Sciences, Hamadan, Iran; 2 Infectious Disease Research Center, Hamadan University of Medical Sciences, Hamadan, Iran; 3 Department of Medical Laboratory Sciences, School of Paramedicine, Hamadan University of Medical Sciences, Hamadan, Iran

**Keywords:** *Escherichia coli*, Blood stream Infections (BSIs), Leukemia, Toxin-antitoxin systems, Virulence factor

## Abstract

**Background:**

One of the most common complications in patients with febrile neutropenia, lymphoma, leukemia, and multiple myeloma is a bloodstream infection (BSI).

**Objective:**

This study aimed to evaluate the antibiotic resistance patterns, virulence factors, biofilm-forming strength, and genetic linkage of *Escherichia coli* strains isolated from bloodstream infections (BSIs) of leukemia patients.

**Methods:**

The study conducted in Iran from June 2021 to December 2022, isolated 67 *E. coli* strains from leukemia patients’ bloodstream infections in hospitals in two different areas. Several techniques including disk diffusion and broth microdilution were used to identify patterns of antibiotic resistance, microtiter plate assay to measure biofilm formation, and PCR to evaluate the prevalence of different genes such as virulence factors, toxin-antitoxin systems, resistance to β-lactams and fluoroquinolone antibiotics of *E. coli* strains. Additionally, the genetic linkage of the isolates was analyzed using the Enterobacterial Repeat Intergenic Consensus Polymerase Chain Reaction (ERIC-PCR) method.

**Results:**

The results showed that higher frequency of BSI caused by *E. coli* in man than female patients, and patients with acute leukemia had a higher frequency of BSI. Ampicillin and Amoxicillin-clavulanic acid showed the highest resistance, while Imipenem was identified as a suitable antibiotic for treating BSIs by *E. coli*. Multidrug-resistant (MDR) phenotypes were present in 22% of the isolates, while 53% of the isolates were ESBL-producing with the *bla*CTX-M gene as the most frequent β-lactamase gene. The fluoroquinolone resistance genes *qnr*B and *qnr*S were present in 50% and 28% of the isolates, respectively. More than 80% of the isolates showed the ability to form biofilms. The *tra*T gene was more frequent than other virulence genes. The toxin-antitoxin system genes (*maz*F, *ccd*AB, and *rel*B) showed a comparable frequency. The genetic diversity was detected in *E. coli* isolates.

**Conclusion:**

Our results demonstrate that highly diverse, resistant and pathogenic *E. coli* clones are circulating among leukemia patients in Iranian hospitals. More attention should be paid to the treatment and management of *E. coli* bloodstream infections in patients with leukemia.

**Supplementary Information:**

The online version contains supplementary material available at 10.1186/s12866-023-03081-8.

## Background

Bloodstream infections (BSIs) are more common in patients with blood cancers such as leukemia and lymphoma and in stem cell transplant recipients [1–4]. The most common risk factor for severe BSI is neutropenia due to cytotoxic chemotherapy [5]. The incidence of Gram-positive blood infections (BSIs) has increased over the past two decades. However, Gram-negative BSI still accounts for approximately 50% of cases and the most common cause is *Escherichia coli* [6]. BSI is associated with a mortality rate of 12–42% [7, 8]. *E. coli* is the most common bacterial species in the human fecal flora due to its ability to colonize the human gastrointestinal tract. Therefore, it is not surprising that it is also the more common cause of Gram-negative BSI in neutropenic patients [9–12]. Extraenteropathogenic *Escherichia coli* (ExPEC) are an isolates that can enter the bloodstream and survive and invade the host [13]. ExPEC strains contain numerous virulence factors (VFs) that allow bacterial cells to colonize. Virulence factors are encoded on bacterial chromosomes, usually located within pathogenicity islands (PAIs), or on plasmids [14]. Several *E. coli* VFs are associated with BSI. Examples of these VFs include adhesions, the iron uptake system, Defense mechanisms against the host, and toxins [15, 16]. However, predicting severity or primary outcome based on bacterial VFs alone is not entirely accurate, and consideration of host determinants, including underlying disease, makes these predictions more acceptable [17]. Treatment of infections caused by ExPEC strains faces serious challenges as strains emerge that are resistant to initial and even final treatments [18].

*E. coli* also has genetic factors known as the toxin-antitoxin (TA) system. *E. coli* contains TA systems, such as MazEF, chpBIK, relBE, yefM-yoeB, dinJ-yafQ, and the mazEF and relBE systems have been studied more than other systems. Toxin-antitoxin (TA) systems are present in bacteria and play an important role in the spread and development of antibiotic resistance through the maintenance of multidrug-resistance plasmids and formation the persister cells, antiphagy defense, and biofilm formation [19].

Antibiotic-resistant Gram-negative and Gram-positive bacteria are now suspected of causing infections in immunocompromised individuals. Enterobacteriaceae that produce extended-spectrum β-lactamases (ESBLs) are elevated in neutropenic cancer patients. The percentage of *Klebsiella pneumoniae* strains producing ESBLs was over 50% in some series, but has been reported to be 11–69% in *E. coli*. *E. coli* can develop resistance to β-lactam antibiotics primarily through the production of ESBLs, although ESBL-encoding plasmids contain resistance genes to other classes of antibiotics. Often Early identification of patients at risk of infection with ESBL-producing bacteria is necessary to guide empirical treatment [20]. Susceptible patients, such as leukemia patients, may be at risk of recurrent infection with *E. coli* strains persisting in the hospital setting. Based on current trends, the risk of recurrent *E. coli* bacteremia, albeit low risk, may be increasing due to the spread of antibiotic-resistant *E. coli* [21].

Molecular typing is a valuable tool in epidemiological research, as it helps trace the origin of infectious agents and determine evolutionary relationships between isolates from various sources. The use of molecular typing methods is crucial for investigating correlations between bacterial strains.

The aim of this study was to evaluate antibiotic resistance patterns, antitoxin systems, virulence factors, biofilm-forming strength, and genetic linkage of the *E. coli* strains isolated from BSI of leukemia patients at two different cancer centers in Iran.

## Methods

### Bacterial isolates

In this cross-sectional study, blood cultures of leukemia patients from June 2021 to December 2022 were obtained from two Iranian medical centers: Hematology-oncology research center in Tehran and 150 bed cancer hospital with an outpatient clinic in Rasht (Northern Iran). Blood samples were cultured in the microbiology laboratory and *E. coli* isolates were identified and confirmed using standard microbiological and biochemical tests [22]. Demographic information such as age, sex, and leukemia type were collected from all leukemia patients.

### Antimicrobial susceptibility

The susceptibility of *E. coli* strains to Imipenem (10 µg), Ceftazidime (30 µg), Ceftriaxone (30 µg), cefotaxime (30 µg), Cefepime (30 µg), Cefixime (30 µg), ciprofloxacin (5 µg), levofloxacin (30 µg), Amikacin (30 µg), Ampicillin (10 µg), Gentamicin (10 µg), Amoxicillin / Clavulanic acid (20 µg), Aztreonam (30 µg), Kanamycin A(30 µg), Trimethoprim/sulfamethoxazole (75 µg), Tetracycline (30 µg) and Nalidixic acid (30 µg) (Mast, UK) were evaluated using the Kirby-Bauer disc diffusion method, based on the Clinical Laboratory Standards Institute (CLSI 2021) guidelines [23]. The minimum inhibitory concentrations (MICs) of Imipenem, Ceftriaxone and Ciprofloxacin (Sigma Aldrich, St. Louis, MO, USA) were determined by broth microdilution method according to CLSI guidelines [23]. Quality control was performed using *E. coli* strain ATCC 25922. Strains resistant to at least one drug of 3 or more antibiotics were considered multi-drug resistant (MDR) strains [24].

### ESBL production testing

Phenotypic detection of ESBL was performed using the double-disc synergy test (DDST) according to CLSI guidelines. This test was performed on Muller Hinton agar with a 30 µg disk of cefotaxime (CTX) along with Cefotaxime –clavunic acid (CA) and 30 µg disc Ceftazidime (CAZ) along with and a disc of Ceftazidime -clavulanate (containing 10 µg of clavulanate) were positioned at a distance of 30 mm (center to center). The test was considered as positive when a decreased susceptibility to Cefotaxime and Ceftazidime were accompanied by significant increase in the inhibitory zone of Cefotaxime and Ceftazidime in front of the disks containing clavulanic acid [23].

### Toxin-antitoxin and virulence factor genes detection

Genomic DNA of *E. coli* strains was extracted from freshly cultured colonies, by boiling method. All strains of *E. coli* were subjected to gene identification of the toxin-antitoxin system, including *mazF*, *relE*, *hipA*, *mqsR*, and *ccdB* by PCR using specific primers (Metabion, Germany) described previously [25, 26], as detailed in Supplementary file 1. PCR reactions were prepared with a total volume of 25 µl. The reaction mixture contained 10 µl of the master mix (Ampliqon, Denmark), 0.5 µl of each primer (10 pmol), 2 µl of the DNA template, and 12 µl of distilled water. The PCR reaction consisted of an initial denaturation step at 95 °C for 5 min, followed by 35 cycles of 60 s at 94 °C, an annealing step as described in Table 1, extension step at 72 °C for 30 s and a final extension step at 72 °C for 10 min. Genes encoding *E. coli* virulence factors, including *hly*A (hemolysin), *iut*A (aerobactin), *afa* (afimbrial adhesion), *tra*T (serum resistance), were also detected by PCR [27–30]. Primer sequences, product sizes, and annealing temperatures of these genes are presented in Supplementary file 1. PCR of virulence genes was performed under conditions of initial denaturation 2 min at 94ºC and 30 cycles including denaturation at 94 °C for 60 s, extension at 72ºC for 90 s, and final extension at 72ºC for 5 min. PCR products were visible after electrophoresis on a 1% agarose gel (Supplementary files 2 and 3).

**Table 1:** Coexistence of ESBL-encoding genes and plasmid-mediated quinolone resistance genes among 67 *E. coli* strains isolated from leukemia patients

Patterns	Frequency (%)
*bla*_CTX−M_ and *qnr*B	**(16/19): 84.2%**
*bla*_TEM_ and *qnr*B	**(10/19): 52.6%**
*bla*_SHV_ and *qnr*B	**(7/19): 36.8%**
*bla*_OXA−48_ and *qnr*B	**(1/19): 5.2%**
*bla*_CTX−M_ and *qnr*S	**(5/7): 71.5%**
*bla*_SHV_ and *qnr*S	**(5/7): 71.5%**
*bla*_TEM_ and *qnr*S	**(2/7): 28.5%**
*bla*_OXA−48_ and *qnr*S	**(1/7): 14.2%**

### Detection of antimicrobial resistance genes

After identification of antibiotic resistance patterns of *E. coli* strains, the β-lactamase-encoding genes of the *bla*_CTX−M_, *bla*_TEM_, *bla*_SHV_, *bla*_OXA−48,_ and *bla*_NDM_ genes, as well as genes encoding fluoroquinolone resistance including *qnr*A, *qnr*B, *qnr*S were detected by PCR. The PCR reactions were prepared in a total volume of 25 µl. Primer sequences, product sizes, and annealing temperatures of these genes are presented in Supplementary file 4. PCR reaction was performed in a thermocycler (Eppendorf, Germany) and 35 cycles were performed for each reaction, The following reaction parameters were used for β-lactamase and quinolone resistance genes respectively: initial denaturation at 94 °C for 30s min followed by 35 cycles including denaturation at 94 °C for 30 s, and extension at 72 °C for 60 s, final extension at 72 °C for 10 min (for β-lactamase genes) as well as initial denaturation at 94 °C for 5 min, followed by 30 cycles including denaturation at 94 °C for 60 s, and, 72 °C for 60 s, 72 °C for 10 min [30–37]. PCR products were detected after electrophoresis on a 1% agarose gel (Supplementary files 5 and 6).

### Quantitative biofilm assay

Biofilm formation of *E. coli* stains was assayed quantitatively using a microtiter plate with crystal violet procedure as described previously [38]. After completing various steps the optical density (OD) of the wells was read at a wavelength of 595 nm using an ELISA reader. Moreover a positive control using *E*. *coli* ATCC 2273, and negative control wells consisting of 200 µl of uninoculated TSB, were included on each plate. All isolates were classified into the following categories: The cut offs were as follows: sample OD < cOD = non-bioiflm producer; cOD < sample OD ≤ 2*(cOD) = weak biofilm producer; 2*(cOD) < sample OD ≤ 4*(cOD) = moderate biofilm producer; sample OD > 4*(cOD) = strong biofilm producers.

### ERIC-PCR

Genetic relatedness of *all E. coli* isolates was analyzed using ERIC-PCR technique. This procedure was performed using the primers as described previously program [39]. The ERIC-PCR patterns of bands on agarose gel was compared by Gel Compar® II v.4.1 software (Applied Maths BVBA, Sint–Martens–Latem, Belgium).

### Statistical analysis

Statistical analyses were performed using IBM SPSS Statistics for Windows software, version 28. The Chi-square test and Fisher’s exact test was used to evaluate correlations among variables. P-values < 0.05 were considered statistically significant.

## Results

### Demographic information of patients

In this cross-sectional study, 67 *E. coli*-positive blood culture samples from leukemia patients were collected and analyzed. Forty-two strains from Tehran and 25 strains from blood cultures in Rasht were isolated, identified and confirmed. Of these 67 strains, 42 (63%) were isolated from male patients and 25 (37%) were isolated from female patients. Patient ages ranged from 6 months to 81 years, with 23% of patients aged 1 to 14 years. Of the 67 leukemia patients, 36 (54%) had acute myeloid leukemia, 16 (24%) had acute lymphoblastic leukemia, 13 (19%) had chronic myeloid leukemia, and 2 (3%) was diagnosed with chronic lymphocytic leukemia.

### Antibiotic resistance patterns

**Fig. 1:**
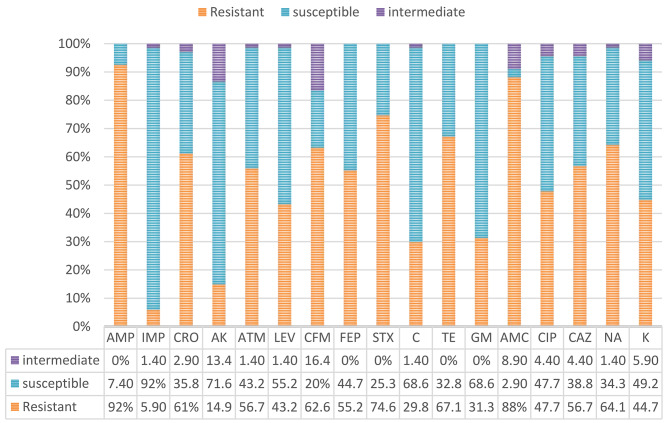
Frequency (%) of antibiotic resistance of *E. coli* strains isolated from Blood cultures of patients with leukemia AMC: Amoxicillin/clavulanic acid, AMP: Ampicillin, ATM: Aztreonam, C: Chloramphenicol, CAZ: Ceftazidime, CFM: Cefixime, FEP: Cefepime, CIP: Ciprofloxacin, LEV: Levofloxacin, GEN: Gentamicin, IMI: Imipenem, SXT: Trimethoprim-sulfamethoxazole, K: Kanamycin, TE: Tetracycline, NA: Nalidixic acid

Figure 1 shows the results of resistance testing against 17 antibiotics. Based on these results, the highest resistance to ampicillin (92%) and Amoxicillin-clavulanic acid (88%) was observed in *E. coli* strains. Resistance to Imipenem was minimal, with 92% of isolates susceptible to Imipenem. More than 50% of isolates were resistant to cephalosporins and over 40% of strains were resistant to fluoroquinolones (ciprofloxacin and levofloxacin). According to the results of the antibiogram, it was found that 15 (22.3%) strains were resistant to at least three antibiotics from three different antibiotic families, which were considered as multidrug resistant (MDR) strains. A total of 36 (53.7%) strains were found to be ESBL-producing strains. In this study, the MIC values of Ciprofloxacin, Ceftriaxone and Imipenem ranged from ≥ 4 µ*g*/ml to ≥ 256 µg/ml, ≥ 16 µg/ml to ≥ 256 µ*g*/ml, and ≥ 64 µ*g*/ml, respectively.

### Prevalence of ESBL and ***qnr*** genes 

PCR results showed that among 36 ESBL producing strains, the frequency of *bla*_CTX−M_ gene (n = 23, 63.8%), was higher than other genes, followed by *bla*_TEM_ (n = 13, 36.1%), *bla*_SHV_ (n = 15, 41.6%), *bla*_NDM-1_ (n = 3, 8.3%) and *bla*_OXA−48_ (n = 3, 8.3%). PCR detection of the *qnr* gene in 32 quinolone-resistant strains revealed that all quinolone-resistant strains were negative for *qnr*A, and (n = 16, 50%) and (n = 9, 28%) of the isolates were positive for *qnr*B and *qnr*S, respectively. The frequency of simultaneous presence of fluoroquinolone and beta-lactam resistance genes was compared in Table 1. *bla*_CTX−M_ and *qnr*B (84.2%) was the most frequent genotype and *bla*_OXA−48_ and *qnr*S showed the lowest frequency.

### Patient characteristics with antibiotic resistance

Table 2 shows the characteristics of leukemia patients classified by ESBL production capacity and resistance to Ciprofloxacin, Ceftriaxone, and Imipenem. The administration of these three antibiotics is important in the treatment of patients with blood infections in the hospitals. In total, the level of resistance to the three mentioned antibiotics and the production of ESBL was higher in patients with acute leukemia. The frequency of ESBL producing strains, resistant to Ciprofloxacin and resistant to Ceftriaxone was higher in patients with acute myeloid leukemia. While in this study, two (50%) Imipenem resistant strains were isolated from acute lymphoblastic leukemia patients.

**Table 2:** Characteristics of 67 *E. coli* strains in bloodstream infections among different types of leukemia based on ESBL production and resistance to Ciprofloxacin, Ceftriaxone, and Imipenem

Hematological malignancy	ESBL (n = 36)	Non-ESBL (n = 31)	Ciprofloxacin R (n = 32)	Ciprofloxacin S (n = 35)	Ceftriaxone R (n = 41)	Ceftriaxone S (n = 24)	Imipenem R (n = 4)	Imipenem S (n = 63)
Acute myeloid leukemia	21(58.3%)	15(48.3%)	15(46.8%)	21(60%)	18(43.9%)	16(66.6%)	1(25%)	35(55.5%)
Acute lymphatic leukemia	8(22.2%)	8(25.6%)	8(25%)	8(25%)	12(29.2%)	4(16.6%)	2(50%)	14(22.2%)
Chronic lymphatic leukemia	5(13.8%)	8(25.6%)	8(25%)	5(14.2%)	9(21.9%)	0	0	12(19%)
Chronic myeloid leukemia	1(2.7%)	1(3.2%)	1(3.1%)	1(2.8%)	2(4.8%)	4(16.6%)	1(25%)	2(3.1%)

### Biofilm phenotypes

As a result of examining the biofilm-forming ability of 67 *E. coli* strains isolated from blood-infected leukemia patients by the microtiter plate method, it was found that 56 strains (83.5%) were capable of forming biofilms. Based on OD, the ability to form biofilms he was classified into four groups: No biofilm formation, strong biofilm, moderate biofilm, and weak biofilm. Of the 67 strains tested, 11 (16.4%) were negative biofilm formers, 25 (37.3%) were weak biofilm formers, 20 (29.8%) were moderate biofilm formers, and 11 were strong biofilm formers.

### Prevalence of virulence factor genes

PCR results of virulence genes of 67 strains of *E. coli* showed a higher rate of *tra*T (77.6%) than other virulence genes, followed by *iut*A (59.7%), *hly*A (55.2%) and *afa* 53.7%. The frequency of simultaneous presence of beta-lactam resistance genes and virulence factor genes was compared in a table in Supplementary file 7. As this table shows, the *bla*_SHV_/*tra*T (93%) and *bla*_CTX−M_ / *tra*T (91%) genotypes were the most frequent, and the next dominant genotype was the *bla*_TEM_ / *tra*T (76%). Genotypes *bla*_OXA_ / *hly*A, *bla*_OXA_/ *iut*A and *bla*_OXA_/ *afa* showed the lowest (33%) frequency.

### Prevalence of toxin-anti toxin genes

**Fig. 2:**
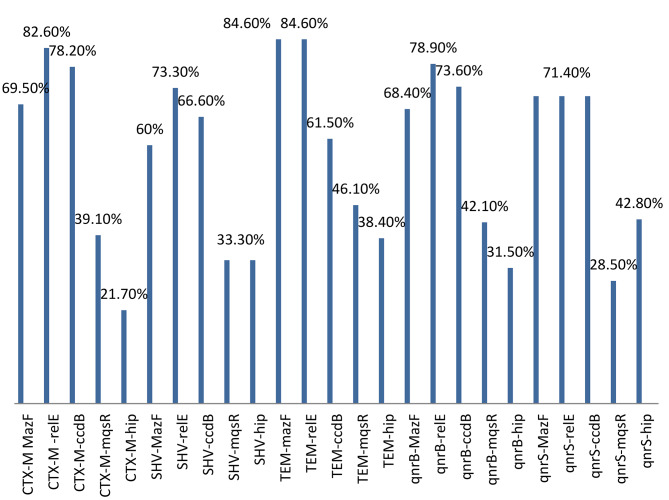
Frequency (%) of coexistence of ESBL-encoding genes, quinolone resistance genes and toxin-antitoxin system genes among 67 *E. coli* strains isolated from blood cultures of patients with leukemia

The results showed that, of the 67 *E. coli* strains, 48(71.6%), 47(70%), 52(77.6%), 24(35.8%) and 21(31.3%) were positive for *maz*E, *ccd*AB, *rel*B, *mqs*R and *hip*A genes, respectively. Therefore, among the genes of the toxin-antitoxin system, the frequency of the *rel*E gene was the highest and the frequency of the *hip*A gene was the lowest. The frequency of simultaneous presence of fluoroquinolone and beta-lactam resistance genes and toxin-antitoxin genes was compared in Fig. 2. As Fig. 2 shows bla_CTXM_/*rel*E, *bla*_TEM_/ *maz*F and *bla*_TEM_/ *rel*E were the most frequent genotypes (≥ 80%) and *bla*_CTXM_-*hip*A was the lowest frequent genotype among ESBL genes positive strains and *qnr*S/relE, *qnr*S/*maz*F, *qnr*S/*ccd*B were the most frequent (≥ 70%) genotypes and *qnr*B/*rel*E was the lowest frequent genotype among *qnr* positive gene strains.

### Genetic linkages of ***E. coli*** strains

**Fig. 3:**
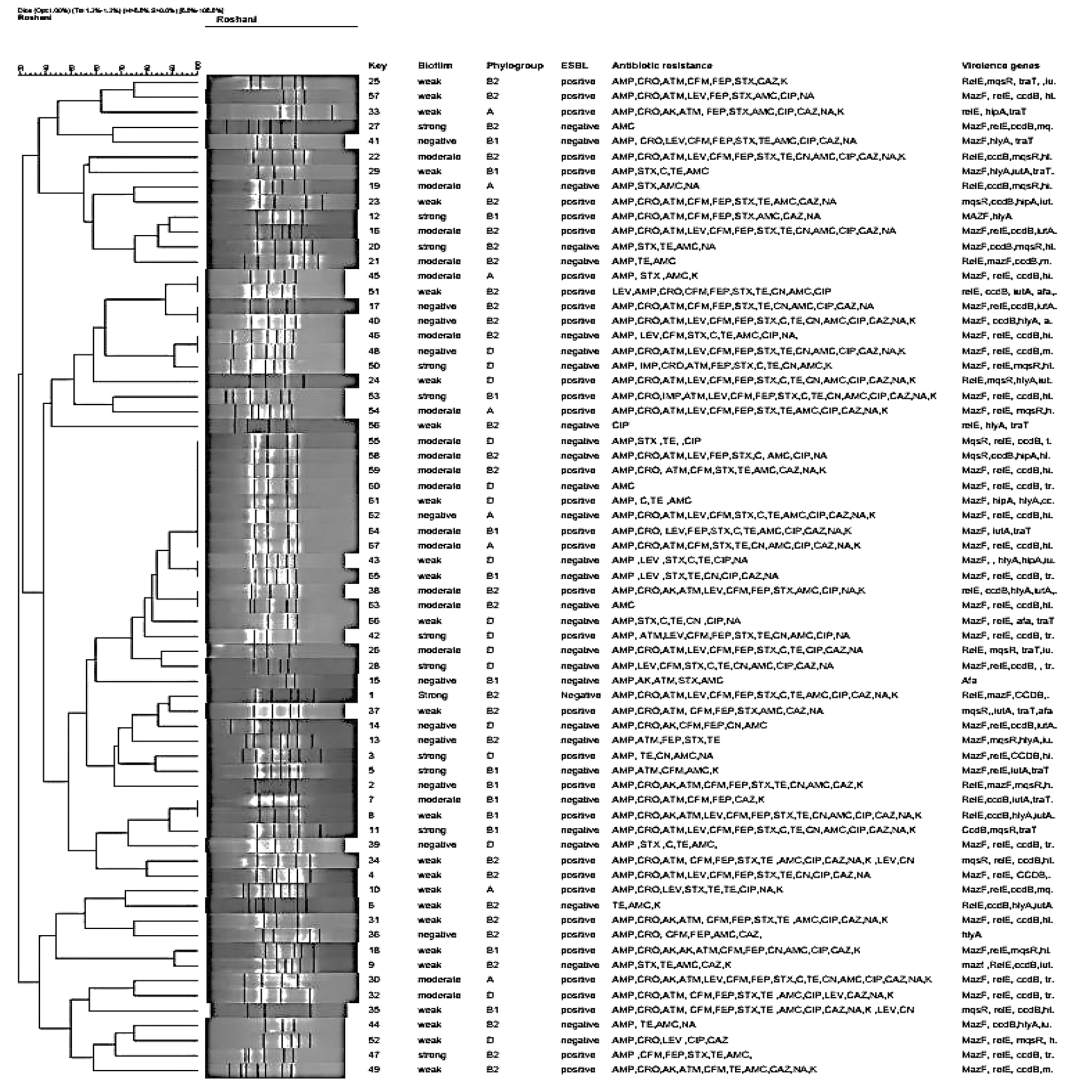
Dendrogram of ERIC-types of 67 *E. coli* strains isolated from blood cultures of patients with leukemia. Biofilm formation strength, presence of ESBL strains, patterns antibiotic resistance, virulence factor gene and toxin-antitoxin genes in different ERIC types were compared. Phylogenetic groups of *E. coli* in this figure was not discussed in this study

Analysis of ERIC-PCR results showed ≥ 80–100% similarity among *E. coli* isolates (Fig. 3). Genetic diversity was established among 67 isolates by detecting 47 different ERIC profiles with a similarity cutoff of ≥ 95%. In total, 47 different ERIC profiles, including 8 common types and 39 single types (including one isolate) was detected among 67 isolates. Out of these eight common types, seven common types included two strains and one common type included 14 different strains.

### Statically analysis results

The results of the statistical analysis of the data showed that there is no significant relationship between the different variables of this study (P value > 0.05).

## Discussion

There have been limited studies conducted in Iran regarding blood infections in leukemia patients. This cross-sectional study aims to provide an overview of the epidemiology of bloodstream infections caused by *E. coli* in patients with hematologic malignancies in two different centers in Iran. The key findings of the study include: (1) a higher occurrence of *E. coli* bloodstream infections in male patients compared to female patients, (2) a higher occurrence of *E. coli* bloodstream infections in patients with acute leukemia, (3) significant resistance to ampicillin and amoxicillin-clavulanic acid, (4) Imipenem was an effective antibiotic for treating *E. coli* bloodstream infections, (5) the presence of multidrug-resistant phenotypes in 22% of isolates, (6) 53% of isolates producing ESBLs with the *bla*_CTX−M_ gene was the most common, (7) the presence of flouroquinolone resistance genes *qnr*B and *qnr*S in 50% and 28% of isolates, respectively, (8) over 80% of isolates having the ability to form biofilms, (9) a higher frequency of the *tra*T gene compared to other virulence genes, with varying frequencies of different virulence genes and the most common genotypes were *bla*_SHV_/*tra*T and *bla*_CTX−M_/*tra*T, (10) a high prevalence of toxin-antitoxin system genes including *maz*E, *ccd*AB, and *rel*B, 11) genetic diversity among *E. coli* strains isolated from bloodstream infections in leukemia patients.

This study offers valuable insights into the epidemiology, resistance patterns, and virulence factors of *E. coli* bloodstream infections in leukemia patients in Iran. Several studies have investigated the prevalence of *E. coli* BSI in leukemia patients. For example, Talcott et al. reported that *E. coli* was the most common cause of BSI in patients with acute myeloid leukemia (AML), accounting for 17% of all BSIs [40]. Another study by Gudiol et al., found that *E. coli* was the second most common cause of BSI in patients with acute lymphoblastic leukemia (ALL), after coagulase-negative staphylococci in a 200-bed university referral cancer center in Barcelona, Spain [41]. A study conducted in Italy found that *E. coli* was the most frequent causative agent of BSI in leukemia patients, accounting for 23% of all cases [42]. A study from Saudi Arabia in 2021 investigated the epidemiology and outcomes of *E. coli* bloodstream infections in adult patients with hematologic malignancies found that *E. coli* was the most common pathogen causing bloodstream infections in these patients, accounting for 32.4% of cases [43].

The occurrence of *E. coli* bloodstream infections in individuals with leukemia can differ based on several factors, including the kind of leukemia, the progression of the illness, the age of the patient, and the existence of other medical conditions. For example, a study from Korea found that the prevalence of *E. coli* BSI was higher in patients with acute myeloid leukemia (AML) than in those with acute lymphoblastic leukemia (ALL) [44]. Another study from Italy reported that the presence of underlying diseases, such as diabetes and chronic obstructive pulmonary disease (COPD), was associated with a higher risk of *E. coli* BSI in leukemia patients [12].

Comparable to other studies, we found that *E. coli* was a commonly isolated pathogen in blood infections among patients with acute leukemia, with 54% of patients having acute myeloid leukemia (AML). The fact that 22% of isolates were from patients with chronic leukemia, and that the male to female ratio of patients suffering from bloodstream infections caused by *E. coli* was 1.68 to 1, suggests that there is a need for further investigation into the risk factors that contribute to bloodstream infections in these patients. Although it has been proven that patients with leukemia are at an increased risk of developing bloodstream infections due to a weakened immune system caused by the cancer itself and the treatment regimens used to manage the disease. Common risk factors for bloodstream infections in patients with acute leukemia include neutropenia, chemotherapy, central venous catheterization, hospitalization, prior antibiotic use, age, and the presence of other medical conditions. Close monitoring and appropriate infection prevention measures are critical in reducing the risk of infections in this patient population.

Antibiotic resistance is a significant concern in the management of bloodstream infections caused by *E. coli* in patients with leukemia or hematologic malignancies. In our study, we found that 22% of isolates were MDR defined as resistance to three or more classes of antimicrobial agents and 53% were ESBL-producing. The presence of the *bla*CTX-M gene was most commonly associated with β-lactamase production, and over 40% of strains were found to be resistant to fluoroquinolone antibiotics.

Multiple studies have investigated the prevalence of antibiotic resistance, multi-drug resistance (MDR), and extended-spectrum β-lactamase (ESBL)-producing strains of *E. coli* in these patient populations. One study conducted in Italy found that 35.7% of *E. coli* bloodstream isolates from patients with hematologic malignancies were resistant to fluoroquinolones, and 20.4% were MDR [12]. Another study from Turkey reported that 64.6% of *E. coli* bloodstream isolates from patients with hematologic malignancies were ESBL producers [45]. In a study from India, the prevalence of ESBL-producing *E. coli* bloodstream infections in patients with hematologic malignancies was found to be 60.2% [46]. A study in 2021 investigated the prevalence and characteristics of ESBL-producing *E. coli* bloodstream infections in patients with hematologic malignancies in China [47]. This study found that ESBL-producing *E. coli* accounted for 24.2% of *E. coli* bloodstream infections in these patients, and like our findings the most common ESBL gene was CTX-M. 58.6% of the strains were ESBL producers [47]. A study from Italy showed that the most common ESBL gene detected was CTX-M, which was found in 85.7% of the ESBL-producing strains. Other ESBL genes detected included TEM and SHV [48]. The high prevalence of ESBL genes in *E. coli* isolates from leukemia patients underscores the need for appropriate antibiotic stewardship and infection control measures to prevent the spread of these multidrug-resistant pathogens. It also highlights the importance of careful use of antibiotics, particularly broad-spectrum antibiotics, in these patients.

In our study Imipenem recognized as a suitable antibiotic to treat BSI in leukemia patients, although resistance to this antibiotic was observed in 5.9% of cases, and caution should be taken in prescribing this antibiotic to these patients so as not to cause more resistance. A study in 2022 investigated the prevalence and risk factors for carbapenem-resistant *E. coli* bloodstream infections in patients with hematologic malignancies in Japan [49]. The study found that the prevalence of carbapenem-resistant *E. coli* bloodstream infections was 2.3%, and that prior exposure to carbapenems and recent hospitalization were significant risk factors. Napolitano et al. found that 14.7% of *E. coli* isolated from patients with hematological malignancies were resistant to imipenem in Italy [48]. A study conducted in Brazil performed genomic analysis of carbapenem-resistant *E. coli* isolates causing bloodstream infections in patients with hematological malignancies. This study found that 9.1% of *E. coli* isolates were resistant to Imipenem [50]. In a study conducted in Iran, the molecular characteristics of carbapenem-resistant *E. coli* strains isolated from patients with hematological malignancies were analyzed. The study revealed a higher prevalence of resistance to Imipenem (33.3%) in *E. coli* isolates compared to our findings [51]. This level of resistance to carbapenems is significant and concerning.

The emergence of Imipenem-resistant *E. coli* strains in patients with leukemia or hematologic malignancies is a significant concern, as these patients are often immunocompromised and more susceptible to severe infections. Regular surveillance of antimicrobial resistance patterns and appropriate infection control measures are crucial in managing these infections.

Overall, the problem of Imipenem resistance in *E. coli* strains causing bloodstream infections in patients with leukemia or hematologic malignancies requires a multifaceted approach that includes a combination of infection control measures, antimicrobial stewardship, and the development of alternative therapies.

Some studies have investigated the prevalence of *qnr*A, *qnr*B, and *qnr*S genes in *E. coli* isolates from leukemia patients. For example, a study in Italy in 2020 found that *qnr* genes were present in 61.2% of the 116 *E. coli* isolates obtained from bloodstream infections in patients with hematological malignancies. The *qnr*S1 gene was the most prevalent, with a prevalence rate of 45.7%. The *qnr*B gene was present in 4.3% of the isolates, while the *qnr*A gene was not detected. Their results were very similar to the results of our study [48]. In a 2019 study conducted in China, it was found that among 72 *E. coli* isolates obtained from leukemia patients, 29.2% carried the *qnr*A gene, 6.9% carried the *qnr*B gene, and 25% carried the *qnr*S gene [52]. However, in the current study, the *qnr*A gene was not detected, while the *qnr*B and *qnr*S genes were present in 50% and 28% of the isolates, respectively.

The detection of *qnr* genes in *E. coli* isolates from leukemia patients is concerning because it suggests that these pathogens may be developing resistance to quinolone antibiotics, which are commonly used to treat infections in these patients. Further research is needed to understand the prevalence and clinical significance of *qnr* genes in leukemia patients.

Biofilm formation is a common virulence factor in many bacterial infections, including bloodstream infections caused by *E. coli*. Biofilms are communities of bacteria that adhere to surfaces and produce a protective extracellular matrix, which can make them more resistant to antibiotics and host immune responses. In our study more than 80% of the isolates showed the ability to form biofilms and 46.2% of *E. coli* isolates were moderate to strong biofilm producers. Several studies have investigated the ability of *E. coli* strains causing bloodstream.

For example a study in Yemen in 2018 found that *E. coli* bloodstream isolates from patients with hematologic malignancies had a high rate of biofilm formation [53]. The study found that 37.3% of *E. coli* isolates were strong biofilm producers, and 36.7% were moderate biofilm producers and the strong biofilm producer’s isolates were more resistant to antibiotics than the weak biofilm producers [53]. Another study investigated the biofilm-forming capacity of *E. coli* isolates from patients with hematological malignancies. The study found that 59.7% of *E. coli* isolates were moderate to strong biofilm producers [54]. A study from Iran in 2019 investigated the relationship between biofilm formation and antibiotic resistance in *E. coli* bloodstream isolates from patients with hematologic malignancies. The study found that biofilm-producing *E. coli* isolates were more likely to be resistant to multiple antibiotics, including beta-lactams, fluoroquinolones, and aminoglycosides [55]. The ability of *E. coli* strains causing bloodstream infections in patients with leukemia or hematologic malignancies to form biofilms is a concern, as it can contribute to treatment failure and disease persistence. Strategies to prevent or disrupt biofilm formation may be important in managing these infections.

*E. coli* strains that cause bloodstream infections (BSIs) in patients with leukemia or hematologic malignancies often carry virulence genes that contribute to their ability to cause disease. In our study several virulence genes were detected. Among these, the *iut*A gene was detected in 59.7% of the strains, followed by *hly*A in 55.2% and *afa* in 53.7%. The *tra*T gene was the most commonly detected virulence gene, being present in 77% of the strains. Notably, the gene associated with iron acquisition was more frequently detected than other virulence genes, which is consistent with our findings and previous studies. Here are some findings from studies that have investigated the prevalence of virulence genes in *E. coli* strains causing BSIs in these patient populations:

Consistent with our study a study, 66 *E. coli* bloodstream isolates from patients with hematologic malignancies in a tertiary care hospital in Romania were tested. The study found that the most common virulence genes detected were *fim*H (89.1%), *tra*T (86.4%), and *iss* (83.6%). These genes are known to be associated with adhesion, iron acquisition, and serum resistance, respectively [56]. Another study investigated the prevalence of virulence genes in *E. coli* isolates from patients with hematological malignancies in Iran [57]. The study found that the most commonly detected virulence genes were *fim*H (95.2%), *tra*T (89.9%), and iss (87.0%), which is consistent with the our and Romanian studies.

The high prevalence of virulence genes in *E. coli* strains causing BSIs in patients with leukemia or hematologic malignancies underscores the importance of appropriate antimicrobial therapy and infection control measures to manage these infections.

Toxin-antitoxin (TA) systems are small genetic modules found in many bacteria that play a role in stress response, persistence, and pathogenesis. Several studies have investigated the prevalence and characteristics of TA system genes in *E. coli* strains causing bloodstream infections (BSIs) in patients with leukemia or hematologic malignancies. In our study we investigated the prevalence of five different TA system genes including *maz*EF, *ccd*AB, *rel*BE, *mqs*R and *hip*A which *rel*BE (77%), *ccd*A (71%) and *maz*EF (70%) were the most frequent TA system gene. A study in 2019 investigated the prevalence and genetic diversity of TA system genes in *E. coli* bloodstream isolates from patients with hematologic malignancies in Iran According to the study, the TA system genes that were most frequently detected were *hig*BA (48.6%), *rel*BE (37.8%), and *maz*EF (35.1%) [58]. However, the prevalence of the TA gene system in our study was found to be higher than these percentages. In a study the prevalence and genetic characteristics of TA system genes in carbapenem-resistant *E. coli* bloodstream isolates from patients with hematologic malignancies was investigated in a Chinese hospital [59]. The study found that the most commonly detected TA system genes were *rel*BE (85.7%), *hig*BA (82.1%), and *maz*EF (75.0%) which is consistent to our finding. Another study investigated the role of TA system genes in the persistence of *E. coli* bloodstream infections in patients with hematologic malignancies in Spanish hospitals [60]. The study found that the presence of certain TA system genes, including *rel*BE and *hig*BA, was associated with increased persistence of infection. Considering that the frequency of *rel*BE gene was also high in our study, this result will be important. These studies suggest that TA system genes are prevalent in *E. coli* strains causing BSIs in patients with leukemia or hematologic malignancies, and may play a role in disease persistence. Further research is needed to fully understand the role of TA systems in the pathogenesis of these infections.

Enterobacterial repetitive intergenic consensus (ERIC)-PCR is a molecular typing method used to differentiate bacterial strains based on variations in the length and sequence of the intergenic regions between conserved ERIC DNA sequences. Several studies have used ERIC-PCR to investigate the genetic diversity and clonal relatedness of *E. coli* strains causing BSIs in patients with leukemia or hematologic malignancies. Results our study and other studies indicated to genetic diversity among *E. coli* strains isolated from BSIs. A study in 2020 used ERIC-PCR to investigate the genetic diversity and clonal relatedness of carbapenem-resistant *E. coli* strains causing BSIs in patients with hematologic malignancies in a Chinese hospital. The study found that the carbapenem-resistant *E. coli* strains were genetically diverse, with no predominant clone identified [59]. However, like our study, these studies did identify several clusters of closely related isolates, suggesting that clonal transmission may have contributed to BSIs. These studies suggest that ERIC-PCR can be a useful tool for investigating the genetic diversity and clonal relatedness of *E. coli* strains causing BSIs in patients with leukemia or hematologic malignancies. However, further research is needed to fully understand the transmission dynamics and pathogenicity of these strains.

### Limitations

Our study had several limitations that affected the scope and depth of our investigation. Firstly, due to the COVID-19 pandemic conditions, we could only collect blood cultures that were positive for *E. coli* and were unable to investigate the frequency of *E. coli* compared to other pathogens in bloodstream infections. Additionally, the study design did not allow us to explore risk factors for bloodstream infections beyond age, sex, and type of leukemia, and our focus was primarily on microbiological aspects. The hospitals from which we obtained our data did not provide additional patient information, further limiting our analysis. Furthermore, we encountered difficulties due to the high cost of materials and tools needed for certain assays, which also restricted the scope of our study. Finally, the overall cost of materials and tools, coupled with the lack of proper cooperation from hospitals, remained significant challenges that we had to overcome in conducting this study.

## Conclusion

Our study highlight the ongoing importance of monitoring the prevalence and characteristics of *E. coli* bloodstream infections in patients with leukemia or hematologic malignancies, in order to inform appropriate antimicrobial therapy and infection control measures. Overall, the patterns of antimicrobial resistance among *E. coli* strains causing bloodstream infections in patients with leukemia or hematologic malignancies can vary depending on the geographic region, patient population, and other factors. It’s important to regularly monitor antimicrobial resistance patterns and adjust treatment strategies accordingly to improve patient outcomes. Further research is needed to fully understand the relationship between *E. coli* biofilm formation ability, virulence factors and toxin-antitoxin system genes and clinical outcomes in these patient populations.

### Electronic supplementary material

Below is the link to the electronic supplementary material.

Supplementary Material 1

Supplementary Material 2

Supplementary Material 3

Supplementary Material 4

Supplementary Material 5

## Data Availability

All the information supporting our conclusions and appropriate references are included in the manuscript. The datasets used and analyzed in the current study are also available from the corresponding author.

## Abbreviations

BSI: Blood Stream Infection
CLSI: Clinical Laboratory Standards Institute
*E. coli*: *Escherichia coli*
ERIC: Enterobacterial Repeat Intergenic Consensus
ESBL: Extended-spectrum β-lactamases
MDR: Multi-Drug Resistant
MIC: Minimum inhibitory concentrations
PAI: Pathogenicity Island
PCR: Polymerase Chain Reaction
TA: Toxin-Antitoxin
VF: Virulence Factor

## References

[1] Huoi C, Vanhems P, Nicolle M-C, Michallet M, Benet T (2013). Incidence of hospital-acquired Pneumonia, bacteraemia and urinary tract Infections in patients with haematological malignancies, 2004–2010: a surveillance-based study. PLoS ONE.

[2] De Naurois J, Novitzky-Basso I, Gill M, Marti FM, Cullen M, Roila F (2010). Management of febrile neutropenia: ESMO clinical practice guidelines. Ann Oncol.

[3] Song Y, Gyarmati P (2019). Bacterial translocation in acute lymphocytic Leukemia. PLoS ONE.

[4] Gyarmati P, Kjellander C, Aust C, Song Y, Öhrmalm L, Giske C (2016). Metagenomic analysis of bloodstream Infections in patients with acute Leukemia and therapy-induced neutropenia. Sci Rep.

[5] Trecarichi EM, Tumbarello M (2014). Antimicrobial-resistant Gram-negative bacteria in febrile neutropenic patients with cancer: current epidemiology and clinical impact. Curr Opin Infect Dis.

[6] Erdem I, Ozgultekin A, Inan AS, Engin DO, Akcay SS, Turan G, Dincer E, Oguzoglu N, Goktas P (2009). Bloodstream Infections in a medical–surgical intensive care unit: incidence, aetiology, antimicrobial resistance patterns of Gram-positive and Gram-negative bacteria. Clinl Microbiol Infect.

[7] Tumbarello M, Sanguinetti M, Montuori E, Trecarichi EM, Posteraro B, Fiori B (2007). Predictors of mortality in patients with bloodstream Infections caused by extended-spectrum-β-lactamase-producing Enterobacteriaceae: importance of inadequate initial antimicrobial treatment. Antimicrob Agent Chemother.

[8] Tumbarello M, Spanu T, Caira M, Trecarichi EM, Laurenti L, Montuori E (2009). Factors associated with mortality in bacteremic patients with hematologic malignancies. Diagn Microbiol Infecti Dis.

[9] Mikulska M, Viscoli C, Orasch C, Livermore D, Averbuch D, Cordonnier C et al. e Fourth European Conference on Infections in Leukemia Group (ECIL-4), a joint venture of EBMT, EORTC, ICHS, ELN and ESGICH/ESCMID. Aetiology and resistance in bacteraemias among adult and paediatric haematology and cancer patients. J Infect. 2013;68:321 – 31. 10.1016/j.jinf.2013.12.006 24370562

[10] Rolston KV, Kapadia M, Tarrand J, Coyle E, Prince RA (2013). Spectrum of gram-positive bacteraemia and in vitro activities of daptomycin, linezolid and Vancomycin against organisms isolated from cancer patients. Int J Antimicrob Agents.

[11] Cattaneo C, Quaresmini G, Casari S, Capucci M, Micheletti M, Borlenghi E (2008). Recent changes in bacterial epidemiology and the emergence of fluoroquinolone-resistant Escherichia coli among patients with haematological malignancies: results of a prospective study on 823 patients at a single institution. J Antimicrob Chemother.

[12] Trecarichi EM, Tumbarello M, Spanu T, Caira M, Fianchi L, Chiusolo P (2009). Incidence and clinical impact of extended-spectrum-β-lactamase (ESBL) production and fluoroquinolone resistance in bloodstream Infections caused by Escherichia coli in patients with hematological malignancies. J Infect.

[13] Russo TA, Johnson JR (2003). Medical and economic impact of extraintestinal Infections due to Escherichia coli: focus on an increasingly important endemic problem. Microbes Infect.

[14] Dale AP, Woodford N (2015). Extra-intestinal pathogenic Escherichia coli (ExPEC): Disease, carriage and clones. J Infect.

[15] Lefort A, Panhard X, Clermont O, Woerther P-L, Branger C, Mentré F (2011). Host factors and portal of entry outweigh bacterial determinants to predict the severity of Escherichia coli bacteremia. J Clini Microbiol.

[16] Mora-Rillo M, Fernández-Romero N, Navarro-San Francisco C, Díez-Sebastián J, Romero-Gómez MP, Arnalich Fernández F (2015). Impact of virulence genes on sepsis severity and survival in Escherichia coli bacteremia. Virulence.

[17] Jauréguy F, Carbonnelle E, Bonacorsi S, Clec’h C, Casassus P, Bingen E (2007). Host and bacterial determinants of initial severity and outcome of Escherichia coli Sepsis. Clin Microbiol Infect.

[18] Calatayud L, Arnan M, Liñares J, Dominguez M, Gudiol C, Carratalà J (2008). Prospective study of fecal colonization by extended-spectrum-β-lactamase-producing Escherichia coli in neutropenic patients with cancer. Antimicrob Agents Chemother.

[19] Jaffe A, Ogura T, Hiraga S (1985). Effects of the ccd function of the F plasmid on bacterial growth. J Bacteriol.

[20] Goudarzi M, Navidinia M (2019). Overview perspective of bacterial strategies of resistance to biocides and antibiotics. Arch Clin Infect Dis.

[21] Samet A, Śledzińska A, Krawczyk B, Hellmann A, Nowicki S, Kur J (2013). Leukemia and risk of recurrent Escherichia coli bacteremia: genotyping implicates E. Coli translocation from the colon to the bloodstream. Eur Clin Microbiol Infect Dis.

[22] Mahon C, Lehman D, Manuselis G. Textbook of Diagnostic Microbiology, Elsevier, Amsterdam, Netherlands, 5th Edidtion edition, 2014.

[23] CLSI. Performance standards for antimicrobial susceptibility testing, 31 St ed. CLSI supplement in M100. Clinical and Laboratory Standards Institute; 2021.

[24] Magiorakos A-P, Srinivasan A, Carey RB, Carmeli Y, Falagas M, Giske C (2012). Multidrug-resistant, extensively drug-resistant and pandrug-resistant bacteria: an international expert proposal for interim standard definitions for acquired resistance. Clin Microbiol Infect.

[25] Karimi S, Ghafourian S, Kalani MT, Jalilian FA, Hemati S, Sadeghifard N. Association between toxin-antitoxin systems and biofilm formation. Jundishapur J Microbiol. 2015;8(1). 10.5812/jjm.14540 PMC4350053 25789127

[26] Narimisa N, Amraei F, Kalani BS, Mohammadzadeh R, Jazi FM (2020). Effects of sub-inhibitory concentrations of antibiotics and oxidative stress on the expression of type II toxin-antitoxin system genes in Klebsiella pneumoniae. J Glob Antimicrob Resist.

[27] Nasser HH, Ismaeel GK, Majeed NM (2018). Gene expression study of pathogenic hemolysin producing E. Coli isolated from cattle by using reverse transcription real-time PCR. Al-Qadisiyah. J Vet Med Sci.

[28] Johnson TJ, Wannemuehler Y, Doetkott C, Johnson SJ, Rosenberger SC, Nolan LK (2008). Identification of minimal predictors of avian pathogenic Escherichia coli virulence for use as a rapid diagnostic tool. J Clin Microbiol.

[29] Bírošová E, Siegfried L, Kmet’ova M, Makara A, Ostró A, Grešová A (2004). Detection of virulence factors in α-haemolytic Escherichia coli strains isolated from various clinical materials. Clin Microbiol Infect.

[30] Melano R, Corso A, Petroni A, Centrón D, Orman B, Pereyra A (2003). Multiple antibiotic-resistance mechanisms including a novel combination of extended-spectrum β-lactamases in a Klebsiella pneumoniae clinical strain isolated in Argentina. J Antimicrob Chemother.

[31] Sharma M, Pathak S, Srivastava P (2013). Prevalence and antibiogram of Extended Spectrum β-Lactamase (ESBL) producing Gram negative bacilli and further molecular characterization of ESBL producing Escherichia coli and Klebsiella spp. J Clin Diagn Res.

[32] Tabar MM, Mirkalantari S, Amoli RI (2016). Detection of ctx-M gene in ESBL-producing E. Coli strains isolated from urinary tract Infection in Semnan. Iran Electron Physician.

[33] Muhammad DM, Elkholy EE-SM, Elmadbouly AA, Montasser KA (2021). Detection of carbapenem-resistant enterobacteriaceae isolates harboring OXA-48 gene in a clinical setting: a two-center-based study. Sci J of Al-Azhar Med Fac Girls.

[34] Seija V, Presentado JCM, Bado I, Ezdra RP, Batista N, Gutierrez C (2015). Sepsis caused by New Delhi metallo-β-lactamase (blaNDM-1) and qnrd-producing Morganella morganii, treated successfully with fosfomycin and meropenem: case report and literature review. Int J Infect Dis.

[35] Robicsek A, Strahilevitz J, Sahm D, Jacoby G, Hooper D (2006). Qnr prevalence in ceftazidime-resistant Enterobacteriaceae isolates from the United States. Antimicrob Agents Chemother.

[36] Robicsek A, Jacoby GA, Hooper DC (2006). The worldwide emergence of plasmid-mediated quinolone resistance. Lancet Infect Dis.

[37] Farshad S, Ranjbar R, Japoni A, Hosseini M, Anvarinejad M, Mohammadzadegan R (2012). Microbial susceptibility, virulence factors, and plasmid profiles of uropathogenic Escherichia coli strains isolated from children in Jahrom, Iran. Arch Iran Med.

[38] Ballash GA, Mollenkopf DF, Diaz-Campos D, van Balen JC, Cianciolo RE, Wittum TE (2022). Pathogenomics and clinical recurrence influence biofilm capacity of Escherichia coli isolated from canine urinary tract Infections. PLoS ONE.

[39] Sedighi P, Zarei O, Karimi K, Taheri M, Karami P, Shokoohizadeh L (2020). Molecular typing of Klebsiella pneumoniae clinical isolates by Enterobacterial repetitive intergenic consensus polymerase chain reaction. Int J Microbiol.

[40] Talcott JA, Finberg R, Mayer RJ, Goldman L, Lee TH. Bloodstream Infections in neutropenic patients with cancer. J Clin Oncol. 28;25:3804–9.

[41] Gudiol C, Bodro M, Simonetti A, Tubau F, González-Barca E, Cisnal M, Domingo-Domenech E, Jiménez L, Carratalà J (2013). Changing aetiology, clinical features, antimicrobial resistance, and outcomes of bloodstream Infection in neutropenic cancer patients. Clin Microbiol Infect.

[42] Trecarichi EM, Pagano L, Candoni A, Pastore D, Cattaneo C, Fanci R, Nosari A, Caira M, Spadea A, Busca A, Vianelli N (2015). Current epidemiology and antimicrobial resistance data for bacterial bloodstream Infections in patients with hematologic malignancies: an Italian multicentre prospective survey. Clin Microbiol Infect.

[43] Alsharif AR, Alshehri SS, Alfehaid LS (2021). Epidemiology and outcomes of Escherichia coli bloodstream Infections in adult patients with hematologic malignancies. Int J Infect Dis.

[44] Park SH, Choi SM, Lee DG (2011). Epidemiology and clinical features of community-onset bacteremia caused by extended-spectrum beta-lactamase-producing Escherichia coli in patients with cancer. Support Care Cancer.

[45] Ozkurt Z, Yilmaz M, Akalin H (2014). Bloodstream Infections in neutropenic cancer patients: results of a multicenter study from Turkey. Antimicrob Resist J Infect Control.

[46] Chakraborty S, Kumar A, Bandyopadhyay M (2015). Characterization of extended-spectrum β-lactamase (ESBL)-producing Escherichia coli obtained from bloodstream Infections in patients with hematological malignancies. Infect Dis.

[47] Yang Y, Shen P, Li X (2021). Prevalence and characteristics of ESBL-producing Escherichia coli bloodstream Infections in patients with hematologic malignancy in China. J Glob Antimicrob Resist.

[48] Napolitano F, Iacobino A, Giordano M (2020). Clonal and resistome analysis of 116 Escherichia coli strains from bloodstream Infections in patients with hematological malignancies. J Glob Antimicrob Resist.

[49] Ito N, Ishiwatari T, Fukuda T (2022). Prevalence and risk factors for carbapenem-resistant Escherichia coli bloodstream Infections in patients with hematologic malignancies: a case-control study in Japan. J Clin Med.

[50] Santos VR, Amaral L, Silva N (2021). Genomic analysis of carbapenem-resistant Escherichia coli isolates from bloodstream Infections in patients with hematological malignancies. Microb Drug Resist.

[51] Jahromi MH, Mirkalantari S, Maleki N (2019). Molecular characteristics of carbapenem-resistant Escherichia coli strains in patients with hematological malignancies. Infect Drug Resist.

[52] Yang HY, Nam YS, Lee HJ (2014). Prevalence of plasmid-mediated quinolone resistance genes among ciprofloxacin-nonsusceptible Escherichia coli and Klebsiella pneumoniae isolated from blood cultures in Korea. Can J Infect Dis Med Microbiol.

[53] Hadi N, Ali YM, Al-Shamahy HA (2018). Biofilm formation and antibiotic resistance in Escherichia coli isolated from patients with hematologic malignancies. J Infect Public Health.

[54] Gao X, Wang L, Zhang Y (2018). Prevalence and characterization of biofilm-producing Escherichia coli isolates from patients with hematological malignancies. J Med Microbiol.

[55] Jahromi MH, Mirkalantari S, Maleki N (2019). Association between biofilm formation and multidrug resistance in Escherichia coli isolated from bloodstream Infections in patients with hematologic malignancies. J Hosp Infect.

[56] Mihaila L, Bolocan A, Gheorghe I (2019). Molecular characterization of virulence genes in Escherichia coli bloodstream isolates from patients with hematologic malignancies. J Immunoass Immunochem.

[57] Mohseni Moghadam F, Soltan Dallal MM, Salehi TZ (2018). Molecular characterization of virulence genes in Escherichia coli isolates from patients with hematological malignancies. J Med Microbiol Infect Dis.

[58] Jahromi MH, Maleki N, Mirkalantari S (2019). Prevalence and genetic diversity of toxin-antitoxin systems in Escherichia coli bloodstream isolates from patients with hematological malignancies. J Med Microbiol.

[59] Zhang H, Liu Y, Wang X, Huang Y, Wang Y, Chen Y (2020). Prevalence and genetic characteristics of toxin–antitoxin systems in carbapenem-resistant Escherichia coli bloodstream isolates from patients with hematologic malignancies. J Glob Antimicrob Resist.

[60] De Majumdar S, Vila J, Moreno-Morales J (2021). Role of toxin-antitoxin systems in the persistence of Escherichia coli bloodstream Infections in patients with hematologic malignancies. J Infect Dis.

