# Supplementary material for: Evaluation of antibiotic resistance, toxin-antitoxin systems, virulence factors, biofilm-forming strength and genetic linkage of Escherichia coli strains isolated from bloodstream infections of leukemia patients
Source: BMC Microbiol. 2023 Nov 4;23:327. doi: 10.1186/s12866-023-03081-8 (PMC10625236; doi:10.1186/s12866-023-03081-8)
Supplement: Supplementary file 1 — Supplementary Material 1 [file 12866_2023_3081_MOESM1_ESM.pdf]

Supplementary file 1: Primer Sequences, Amplicon Sizes, and Annealing Temperature of Toxin-Antitoxin System and Virulence Genes in E. coli Strains Isolated from Leukemia Patients' Blood Cultures.

Target gene	Primer sequence (5′-3′)	Product size (bp)	Annealing Temperature (°C)
mazF	F:ATGGTAAGCCGATACGTACCC<br>R: TGGGGCAACTGTTCCTTT	288	58.5 °C
relE	F: CAAATGGTTCGCCAGAGAGG<br>R: GCGATTCTTGTTGAGTCGCT	136	58.6 °C
hipA	F: GATAAACAGTGCCGTACGCT<br>R: CAAATGGTTCGCCAGAGAGG	156	58.7 °C
ccdB	F:GAGAGAGCCGTTATCGTCTGTT<br>R:TCCCCAGAACATCAGGTTAATG	272	58.7 °C
mqsR	F:ACGCACACCACATACACGTT<br>R:GCCTGGGTCTGTAAACATCCT	194	58.7 °C
hlyA	F:AATGGGAAAAGGAGCATGGC<br>R:GCAAAACCAAGTTGGGTGTG	143	60 °C
iutA	F:GGCTGGACATCATGGGAACTGG<br>R:CGTCGGGAACGGGTAGAATCG	302	58 ˚C
***tra*T**	F:GGTGTGGTGCGATGAGCACAG<br>R:CACGGTTCAGCCATCCCTGAG	290	56 ˚C
afa	F:GCTGGGCAGCAAACTGATAACTCTC<br>R:CATCAAGCTGTTTGTTCGTCCGCCG	750	68 ˚C
