# Supplementary material for: Evaluation of antibiotic resistance, toxin-antitoxin systems, virulence factors, biofilm-forming strength and genetic linkage of Escherichia coli strains isolated from bloodstream infections of leukemia patients
Source: BMC Microbiol. 2023 Nov 4;23:327. doi: 10.1186/s12866-023-03081-8 (PMC10625236; doi:10.1186/s12866-023-03081-8)
Supplement: Supplementary file 2 — Supplementary Material 2 [file 12866_2023_3081_MOESM2_ESM.pdf]

Evaluation of antibiotic resistance, toxin-antitoxin systems, virulence factors, biofilm- forming strength and genetic linkage of *Escherichia coli* strains isolated from bloodstream infections of leukemia patients

Mahdaneh Roshani, Mohammad Taheri, Alireza Goodarzi, Rassoul Yosefimashouf, Leili Shokoohizadeh

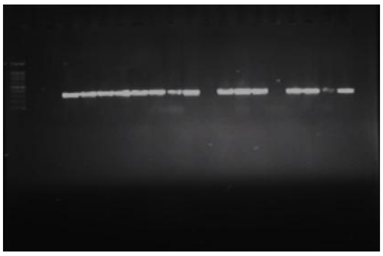

MazF

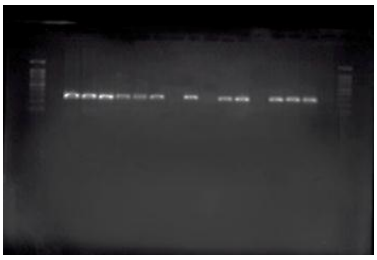

ccdB

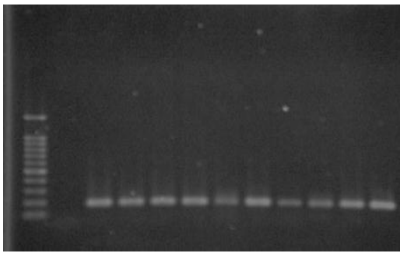

mqsR

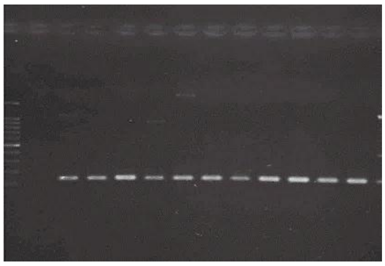

hipA

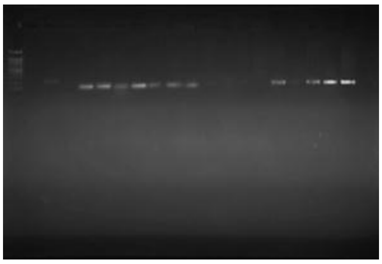

relE

Supplementary file 2: Gel electrophoresis image of toxin-antitoxin genes in *E. coli* strains isolated from leukemia patients' blood cultures: *maz*F: 288 bp, *ccd*B: 272bp, *mqs*R: 194 bp, *hip*A: 196 bp, *rel*E: 136 bp (The cropping in the some gel images are due to the presence of PCR products of other genes with different sizes that have been removed from the gel Images).
