# Supplementary material for: Evaluation of antibiotic resistance, toxin-antitoxin systems, virulence factors, biofilm-forming strength and genetic linkage of Escherichia coli strains isolated from bloodstream infections of leukemia patients
Source: BMC Microbiol. 2023 Nov 4;23:327. doi: 10.1186/s12866-023-03081-8 (PMC10625236; doi:10.1186/s12866-023-03081-8)
Supplement: Supplementary file 3 — Supplementary Material 3 [file 12866_2023_3081_MOESM3_ESM.pdf]

Evaluation of antibiotic resistance, toxin-antitoxin systems, virulence factors, biofilm- forming strength and genetic linkage of *Escherichia coli* strains isolated from bloodstream infections of leukemia patients

Mahdaneh Roshani, Mohammad Taheri, Alireza Goodarzi, Rassoul Yosefimashouf, Leili Shokoohizadeh

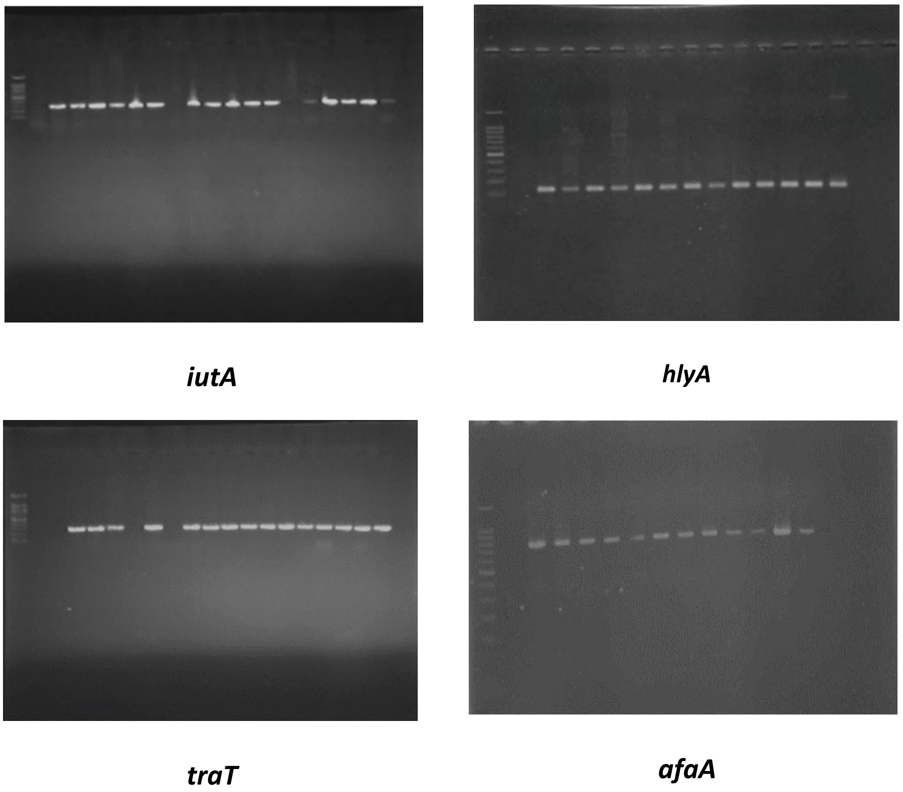

Supplementary file 3: Gel electrophoresis image of virulence factor genes in *E. coli* strains isolated from leukemia patients' blood cultures. *iut*A: 302bp, *hly*A: 143 bp, *tra*T: 290 bp, *afa*A: 750 bp
