# Supplementary material for: Evaluation of antibiotic resistance, toxin-antitoxin systems, virulence factors, biofilm-forming strength and genetic linkage of Escherichia coli strains isolated from bloodstream infections of leukemia patients
Source: BMC Microbiol. 2023 Nov 4;23:327. doi: 10.1186/s12866-023-03081-8 (PMC10625236; doi:10.1186/s12866-023-03081-8)
Supplement: Supplementary file 4 — Supplementary Material 4 [file 12866_2023_3081_MOESM4_ESM.pdf]

Supplementary file 4: Primer Sequences, Amplicon Sizes, and Annealing Temperature of ESBL and *qnr* Genes in *E. coli* Strains Isolated from Leukemia Patients' Blood Cultures

Target gene	Primer sequence (5′-3′)	Product size (bp)	Annealing Temperature (°C)
bla*TEM -F**<br>bla*TEM-R**	TTGGGTGCACGAGTGGGTTA<br>TAATTGTTGCCGGGAAGCTA	500	52℃
bla*SHV-F**<br>bla*SHV-R**	AGGATTGACTGCCTTTTTG<br>ATTTGCTGATTTCGCTCG	392	54℃
bla*CTX-M-F**<br>bla*CTX-M-R**	ACCGCCGATAATTCGCAGAT<br>GATATCGTTGGTGGTGCCATAA	585	55℃
**blaOXA-48F**<br>**OXA-48R**	GCTTGATCGCCCTCGATT<br>GATTTGCTCCGTGGCCGAAA	281	60℃
bla*NDM-1F**<br>bla*NDM-1R**	GGTTTGGCGATCTGGTTTTC3<br>CGGAATGGCTCATCACGATC	621	52℃
qnrA (F)<br>***qnrA (R***	ATTTCTCACGCCAGG ATT TG<br>AT CGG CAA AGG TTA GGT CA	571	55℃
qnrB (F)<br>***qnrB (R***	GATCGTGAAAGCCAGAAAGG<br>ACGATG CCT GGT AGT TGT CC	594	57℃
qnrS (F)<br>***qnrS (R***	ACGACATTCGTCAACTGC AA<br>TAAATTGGCACCCTGTAG GC	388	57℃
