# Supplementary material for: Evaluation of antibiotic resistance, toxin-antitoxin systems, virulence factors, biofilm-forming strength and genetic linkage of Escherichia coli strains isolated from bloodstream infections of leukemia patients
Source: BMC Microbiol. 2023 Nov 4;23:327. doi: 10.1186/s12866-023-03081-8 (PMC10625236; doi:10.1186/s12866-023-03081-8)
Supplement: Supplementary file 5 — Supplementary Material 5 [file 12866_2023_3081_MOESM5_ESM.pdf]

Evaluation of antibiotic resistance, toxin-antitoxin systems, virulence factors, biofilm-forming strength and genetic linkage of *Escherichia coli* strains isolated from bloodstream infections of leukemia patients

Mahdaneh Roshani, Mohammad Taheri, Alireza Goodarzi, Rassoul Yosefimashouf,

Leili Shokoohizadeh

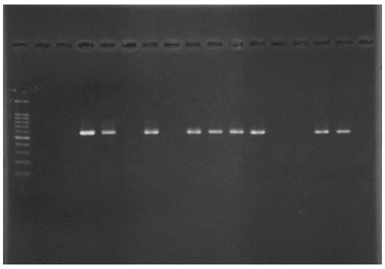

***bla* CTXM**

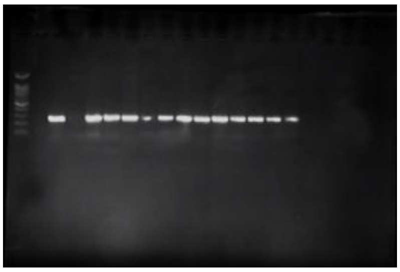

***bla* OXA-48**

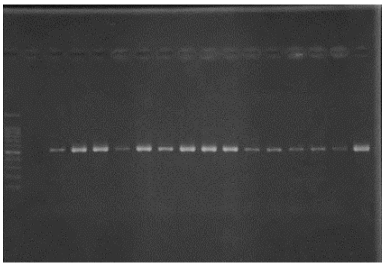

***bla* TEM**

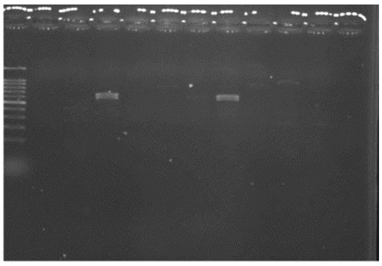

***bla* NDM-1**

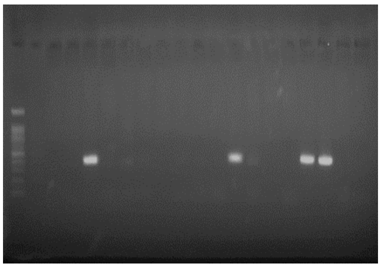

***bla* SHV**

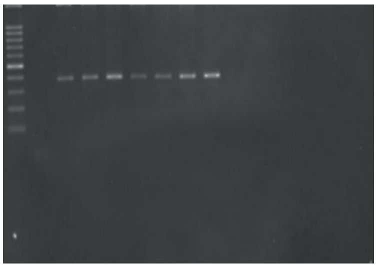

***qnr*S**

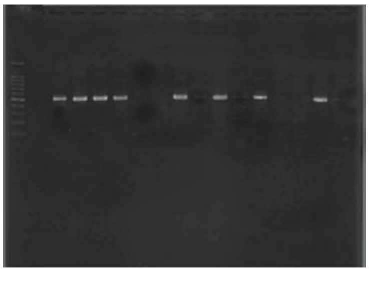

***qnr*A**

Supplementary file 5: Gel electrophoresis image of ESBL and *qnr* genes in *E. coli* strains isolated from leukemia patients' blood Cultures: *bla* $_{\text{CTX-M}}$: 585 bp, *bla* $_{\text{OXA-48}}$: 281 bp, *bla* $_{\text{TEM}}$: 500 bp, *bla* $_{\text{NDM-1}}$: 621 bp, *bla* $_{\text{SHV}}$: 392, *qnr*S: 388 bp, *qnr*A: 572 bp (The difference in the size of the wells and agarose gels is the reason for the use of agarose gel cassettes with different sizes, which were selected according to the needs and number of samples).
